# MiR-3064 in Epicardial Adipose-Derived Exosomes Targets Neuronatin to Regulate Adipogenic Differentiation of Epicardial Adipose Stem Cells

**DOI:** 10.3389/fcvm.2021.709079

**Published:** 2021-08-18

**Authors:** Wenkai Yang, Hanjian Tu, Kai Tang, Haozhong Huang, Shi Ou, Jianguo Wu

**Affiliations:** ^1^Department of Cardiovascular Surgery, Central People's Hospital of Zhanjiang, Zhanjiang, China; ^2^Department of Cardiac Surgery, Shanghai East Hospital, Tongji University School of Medicine, Shanghai, China

**Keywords:** coronary atherosclerotic heart disease, epicardial adipose stem cells, exosomes, miR-3064-5p, neuronatin

## Abstract

**Backgroud:** The metabolism of epicardial adipose tissue (EAT) is closely related to coronary atherosclerotic heart disease (CAHD), but the specific mechanism is not fully understood. In this study, we investigated the effects of EAT microenvironment on adipose metabolism from the viewpoint of EAT-derived exosomes and epicardial adipose stem cells (EASCs).

**Methods:** EAT samples from CAHD patients and non-CAHD patients were collected to obtain exosomes via tissue culture. MiRNA sequencing was performed to analyze differences in miRNA expression in exosomes between groups. Luciferase reporter assay was then performed to verify the miRNA target gene. EAT was digested by collagenase to obtain EASCs, which were induced to mature adipocytes *in vitro*. Immunochemical staining and western blotting were performed to detect protein expression levels.

**Results:** The results showed that CAHD patients had higher levels of EASCs in EAT, and no significant difference in the adipogenic differentiation ability of EASCs was observed between CAHD and non-CAHD patients *in vitro*. This indicates that the EAT microenvironment is a key factor affecting the adipogenic differentiation of EASCs. The EAT-derived exosomes from CAHD patients inhibited adipogenic differentiation of EASCs *in vitro*. Sequencing analysis showed that miR-3064-5p was highly expressed in EAT-derived exosomes in CAHD patients, and its inhibitor could improve the adipogenic differentiation of EASCs. Luciferase reporter assay results showed that the target gene of miR-3064-5p is neuronatin (Nnat). Nnat remained silent in EASCs and was less expressed in EAT of CAHD patients.

**Conclusion:** Abovementioned results suggest that Nnat is the key to regulating the adipogenic differentiation of EASCs, and miR-3064-5p in EAT-derived exosomes can inhibit the expression of Nnat by targeting its mRNA, thereby affecting the adipogenic differentiation of EASCs.

## Introduction

Atherosclerosis (AS) causes ~20 million deaths worldwide each year. AS is a slowly progressive disease with complex pathogenesis, and its exact etiology is still not fully understood. It is now generally believed that AS is a chronic inflammatory disease, closely related to hyperlipidemia, hypertension, diabetes, and genetic factors (1). Therefore, elucidating the pathogenesis of AS and finding new targets and treatments are the key issues in the prevention and treatment of AS.

Epicardial adipose tissue (EAT) is the adipose tissue located between the myocardium and the pericardium, which surrounds and directly contacts the cardiac blood vessels. Owing to its elasticity and compressibility, EAT can protect coronary arteries from excessive distortion caused by arterial pulsation and myocardial contraction (2). EAT, as a local storage site of excess free fatty acids, maintains myocardial energy supply and prevents the toxic effects of high circulating free fatty acids on the myocardium and coronary arteries (3). There is no connective tissue or aponeurotic tissue between EAT and myocardium, indicating a close and strong interaction between them. Unlike pericardial fat, EAT angiogenesis depends on the branches of the coronary arteries, further indicating a close relationship between EAT and myocardial tissue (2). Although not fully elucidated, a growing body of evidence supports that EAT with metabolic disorders promotes the progression of coronary atherosclerotic heart disease (CAHD) (4). Thus, correcting the disordered EAT metabolism may be a potential method for the prevention and treatment of CAHD.

Histological analysis of EAT showed that it is a mixed cell structure, mainly comprising adipose stromal cells, and contains a large number of inflammatory cells including lymphocytes, macrophages, and mast cells (5). Studies have confirmed that EAT is rich in adipose stem cells (ASCs), which express stem cell markers (6). Epicardial adipose stem cells (EASCs) have higher myocardial and angiogenic potential compared with stem cells derived from pericardial and omental adipose tissues (5, 6). However, few studies have analyzed the existence and the functions of EASCs, in both animal and human studies, and the relationship between EASCs and CAHD is also rarely reported. In the preliminary experiments, we found that the abundance of EASCs in EAT of CAHD patients was higher than that in the EAT of non-CAHD patients. We speculate that the abnormal adipogenic differentiation of EASCs may be the cause of the metabolic disorder of EAT in CAHD patients, and have explored EAT-derived exosomes form that perspective in this study.

## Materials and Methods

### Reagents

The antibodies for CD9, CD44, CD81, calnexin, neuronatin (Nnat), and GAPDH were all purchased from Proteintech (Rosemont, IL, USA). The Oil red O stain was commercially obtained from Solarbio (Beijing, CHN). Lipofectamine 2000 was purchased from ThermoFisher (Waltham, MA, USA).

### Sample Acquisition

The patients signed an informed consent form, which was approved by the hospital ethics committee, and the study complied with the Declaration of Helsinki. The patient samples were collected in accordance with the relevant Chinese laws and regulations, and the samples obtained were registered with the relevant government departments. From January 2020 to December 2020, there were 39 planned cardiac surgery patients in total (Table 1). According to clinical diagnosis, the patients were divided into the AS group (*n* = 24) and non-AS group (*n* = 15). During the operation, an EAT biopsy sample (0.5–1.0 g) was collected from the aortic root near the right coronary artery and divided into two parts. One part was put in the sample preservation solution and transported to the laboratory and stored at a low temperature, and the other part was fixed with formaldehyde.

**Table 1:** Characteristics of patients.

	**non-AS group (*n* = 15)**	**AS group(*n* = 24)**	***P*-value**
Age (year)	62.47 ± 2.13	67.13 ± 1.39	0.0636
Male/Female	5/10	16/8	0.0549
Body mass index (kg/m^2^)	24.41 ± 0.49	25.81 ± 0.48	0.0605
Clinical diagnosis			
Rheumatic heart disease	15	–	
Coronary atherosclerotic heart disease	–	24	
Reason for surgery			
Valvular lesion	15	–	
Multivessel coronary stenosis	–	24	

### EASCs Culture

In the biosafety cabinet, fresh EAT samples were washed with PBS, and then digested with 0.075% type I collagenase by shaking at 37°C for 1 h. Following this, the same volume of cell culture medium was added, with repeated blow and mix. After centrifugation, the supernatant and residual fat were discarded to obtain cell sediment. The primary cell culture medium (Procell, Wuhan, CHN) was added to resuspend the cells and inoculated into T25 culture bottle. The cells were cultured in a humidified atmosphere with 5% CO_2_. After 24 h, the medium was discarded to remove the non-adherent cells. The cells were then fed every 3 days, and sub-cultured at 70–80% confluency. CD44 and CD45 were detected in EASCs via immunofluorescence and flow cytometry.

### Exosomes Extraction

In the biosafety cabinet, fresh EAT tissue sample was divided into 2 ×2 × 2 mm tissue blocks, and transferred into the culture bottle. Turning over the culture bottle gently, an appropriate amount of culture medium prepared with exosome-free fetal bovine serum was added to the bottle. After 2 h, the culture bottle was turned back gently and the culture medium slowly covered the tissue blocks which would be cultured in the incubator conventionally. The culture medium was collected regularly to separate the exosomes by high-speed centrifugation, and the morphology of exosomes was observed by transmission electron microscopy (HITACHI, Tokyo, Honshu, JPN).

### Oil Red O Staining

EASCs from CAHD patients or non-CAHD patients were induced differentiation into mature adipocytes using an adipogenic differentiation medium (Procell, Wuhan, HB, CHN) according to the instruction of manufacturer. EAT-derived exosomes were categorized into the non-AS group and AS group, and inoculated with EASCs from CAHD patients at a concentration of 20 μg/mL, which was determined based on preliminary experiments. After 21 days of adipogenic induction, the culture medium was discarded and the cells were stained with oil red O dye as reported previously (7).

### Immunohistochemical Analysis

Briefly, paraffin sections of EAT samples and climbing slices of EASCs were incubated with target antibodies. All sections were photographed at 20 × magnification (Leica Microsystems Inc., IL, USA), and analyzed using the Image Pro Plus 6.0 software (Media Cybernetics, Rockville, MA, USA) accordingly.

### MiRNA Sequencing

The total RNA in exosomes was extracted using the miRNA isolation kit (mirVana, Austin, TX, US), qualified by electrophoresis, and sequenced accordingly. The sequencing reagent was prepared according to user guide of Illumina (San Diego, CA, USA), and the sample was analyzed by an Illumina sequencer. The single-read program was used for single-end sequencing. The sequencing process was controlled by the data collection software provided by Illumina, and real-time data analysis was carried out.

### Quantitative Polymerase Chain Reaction (qPCR) Assay

*in vitro* delivery of miR-3064-5p inhibitor-loaded exosomes to EASCs by incubating in culture medium. After 24 h, the expression of miR-3064-5p in EASCs was detected using qPCR. Briefly, total RNA was reverse transcribed using the MicroRNA Reverse Transcription Kit (Haoqinbio Inc., Shanghai, China) with specialized primers according to the manufacturer's instruction. RNU6 was used as a housekeeping reference. The synthesized first-strand cDNA samples were subjected to qPCR using hsa-miR-3064-5p specific TaqMan primer (Applied Biosystems, Foster City, USA) and TaqMan Universal PCR Master Mix in an ABI Prism 7700 Sequence Detector (ThermoFisher, Waltham, MA, USA). The oligonucleotide primer sequence of Nnat was designed using Primer 5.0 software and GAPDH was used as an internal control. The synthesized first-strand cDNA samples were subjected to qPCR using a SYBR Green PCR Master Mix (Toyobo Bio-Technology, Shanghai, CHN) and the qPCR reaction was also performed on the ABI Prism 7700 Sequence Detector (ThermoFisher).

### Luciferase Reporter Assay

The EASCs were cultured *in vitro* to induce differentiation into mature adipocytes. The reporter plasmid and miRNA mimics were co-transfected into adipocytes using the Lipofectamine 2000 (ThermoFisher, Waltham, MA, USA) transfection reagent, and the Dual-Luciferase Reporter Assay System (Promega, Madison, WI, USA) was used to observe luciferase activity. Furthermore, RT-PCR and western blotting were sequentially performed to further verify mRNA and protein levels.

### Western Blotting

Proteins were extracted from EAT or EASCs using radioimmunoprecipitation assay (RIPA) lysis buffer and size fractionated by SDS polyacrylamide gel electrophoresis. Membranes were incubated with target antibodies at 4°C overnight. Then, membranes were incubated with horseradish peroxidase-conjugated secondary antibodies for 2 h at room temperature and washed with tris-buffered saline and Tween 20. The immune complexes were visualized by enhanced chemiluminescence after washing again, and the band intensity was measured quantitatively and analyzed with the Image J v2.1.4.7 software (National Institutes of Health, Bethesda, MD, USA).

### Statistical Analysis

All data are presented as means ± standard error of the mean and analyzed using SPSS version 20.0 (IBM Corp., Armonk, NY, USA). Two-tailed Student's *t*-tests were performed to compare means between two groups. A *P*-value < 0.05 was considered to be statistically significant.

## Results

### EASCs in EAT of CAHD Patients Are More Abundant Than Those in the EAT of Non-CAHD Patients

**Figure 1:**
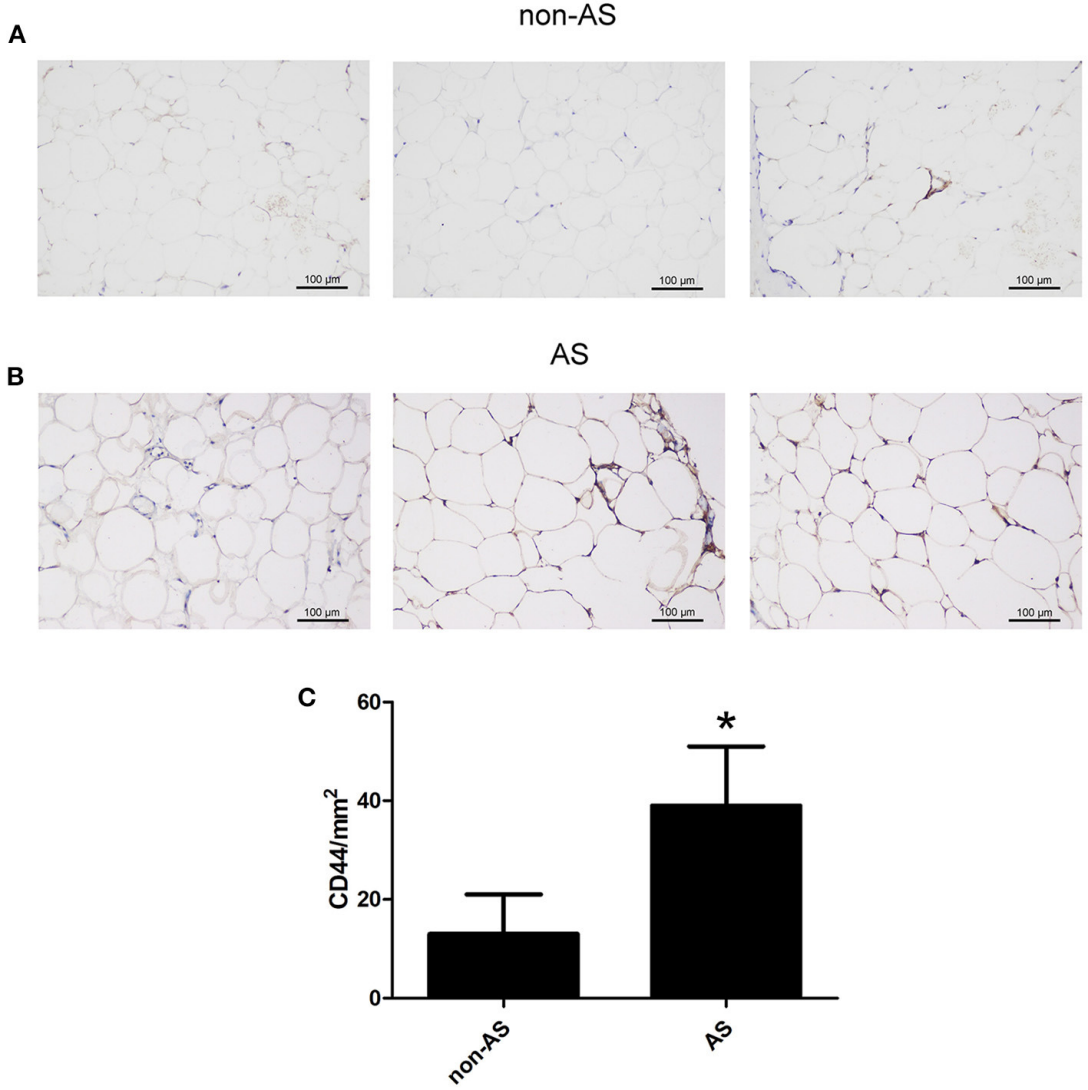
The expression of CD44 in EAT of CAHD or non-CAHD patients. **(A)** The expression level of CD44 in EAT of non-CAHD patients. **(B)** The expression level of CD44 in EAT of CAHD patients. **(C)** The comparison of the number of CD44 positive cells in EAT of CAHD or non-CAHD patients. non-AS: the EAT of non-CAHD patients. AS: the EAT of CAHD patients. *P < 0.05 vs. non-AS.

**Figure 2:**
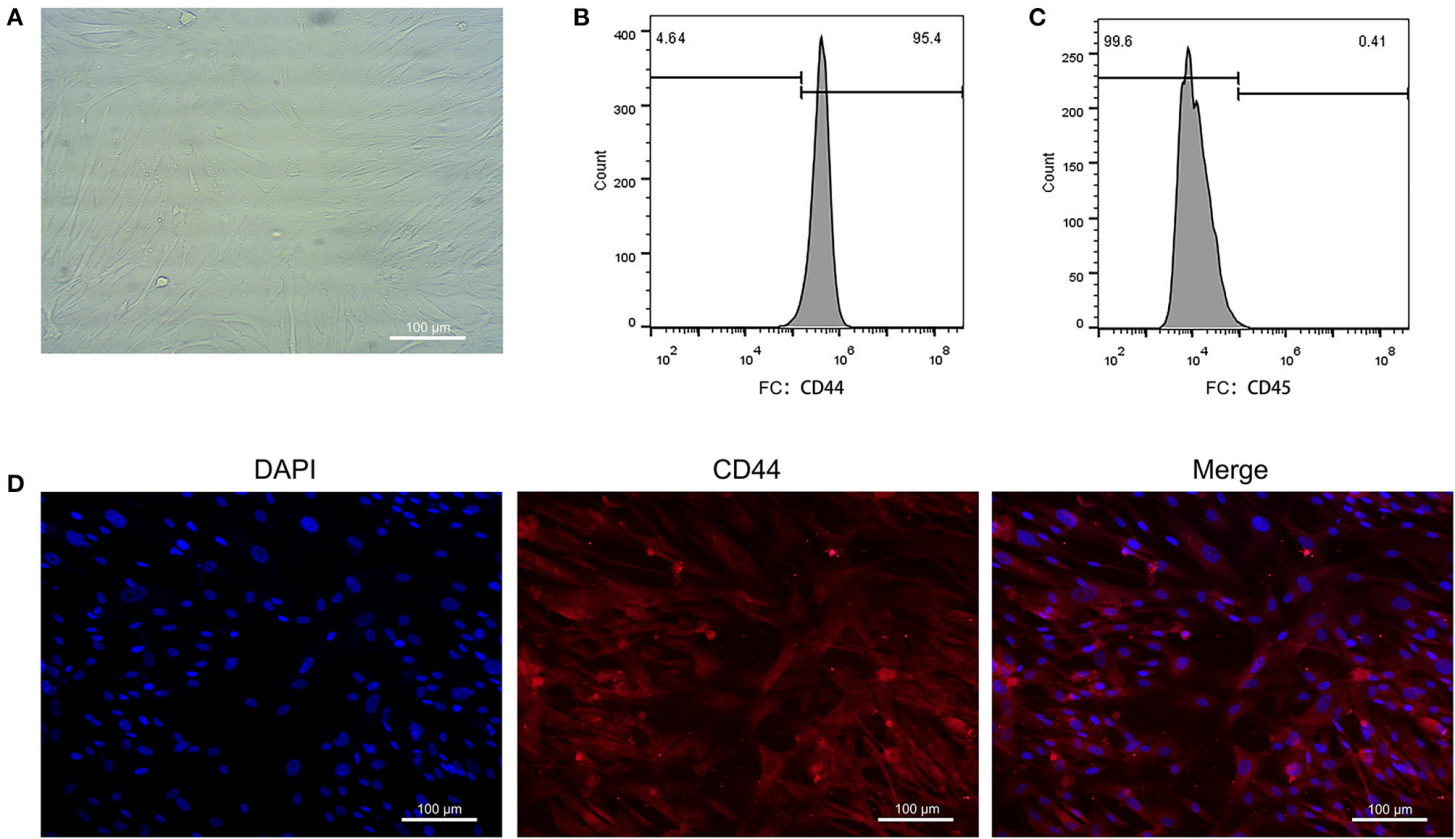
Primary culture of EASCs and its identification. **(A)** Morphology of EASCs under microscope. **(B)** Flow cytometry of CD44 positive cells in EASCs. **(C)** Flow cytometry of CD45 positive cells in EASCs. **(D)** Immunofluorescence staining of CD44 in EASCs climbing slices.

The EAT from CAHD patients (AS group) and non-CAHD patients (non-AS group) were collected to perform immunohistochemical staining for CD44, one of biomarkers of EASCs. As shown in Figure 1, the number of CD44-positive cells in the EAT of CAHD patients was significantly higher than that in the EAT of non-CAHD patients (*P* < 0.05), indicating that CAHD patients show a higher abundance of EASCs than non-CAHD patients. Human EAT was digested with collagenase to screen out adherent cells, and the cell morphology was found to be mesenchyma-like (Figure 2A). The positive rate of anti-CD44 on the cell surface detected by flow cytometry was 95.4% while the CD45 was 0.41% (Figures 2B,C), which was consistent with the characteristics of adipose stem cells. The CD44 antibody was used for immunofluorescence staining of cell slides, showing >90% positive cells (Figure 2D).

### Exosomes Derived From EAT of CAHD Patients Inhibit the Adipogenic Differentiation of EASCs

**Figure 3:**
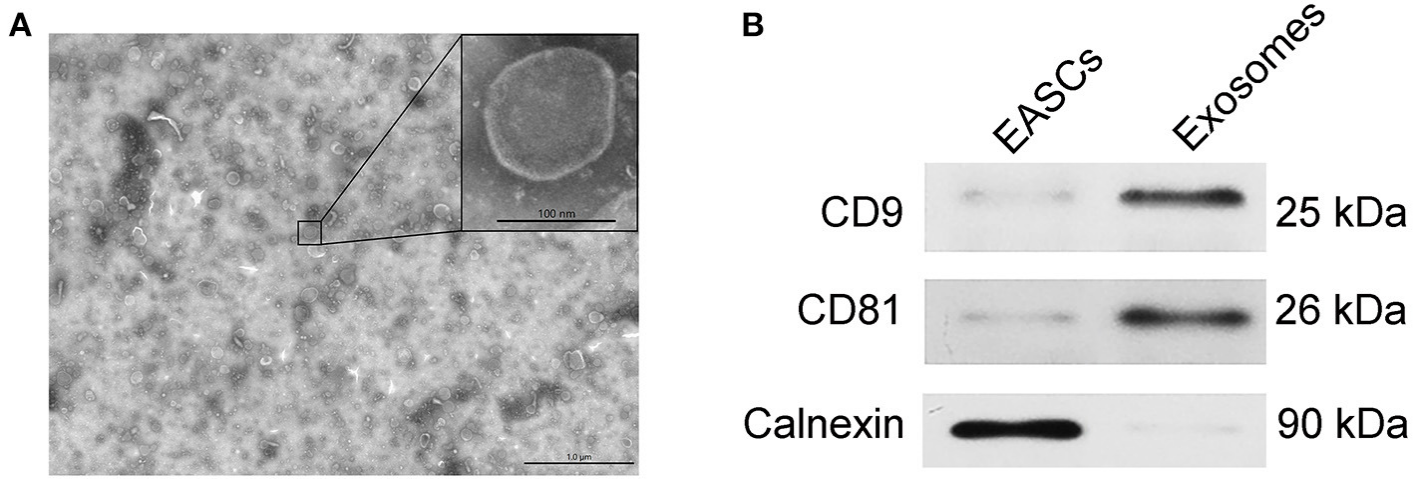
Identification of exosomes from EAT. **(A)** Electron micrographs of exosomes. **(B)** The protein expression levels of CD9, CD81 and calnexin in EAT-derived exosomes.

The EAT tissue blocks were cultured with exosome-free serum *in vitro*, and the exosomes were extracted from the medium. As shown in Figure 3A, vesicle-like structures with obvious lipid bilayers could be observed via electron microscope. The total proteins of vesicles were extract, and the expression levels of the exosome markers CD9 and CD81 and the endoplasmic reticulum-specific molecule Calnexin were detected by Western blotting. Total proteins of EASCs were used as a control. As shown in Figure 3B, the expressions of CD9 and CD81 were detected in the total proteins of vesicles, whereas Calnexin was negatively expressed, suggesting that the vesicle structures are exosomes.

**Figure 4:**
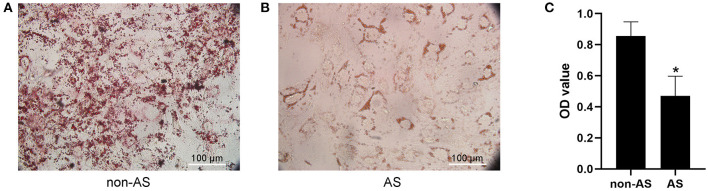
Effect of EAT-derived exosomes on adipogenic differentiation of EASCs. Here the EASCs were derived from the EAT of CAHD patients. **(A)** The representative image of oil red O staining in non-AS group. **(B)** The representative image of oil red O staining in AS group. **(C)** Oil red O was extracted and absorbance was determined spectrophotometrically at 450 nm to quantify adipogenic differentiation. non-AS: the group of EASCs intervened with the EAT-derived exosomes of non-CAHD patients. AS: the group of EASCs intervened with the EAT-derived exosomes of CAHD patients. Data are presented as the means ± standard error of the mean for six independent experiments. ^*^P < 0.05 vs. non-AS.

The adipogenic induction medium was used to induce adipogenic differentiation of EASCs and no significant difference in the level of adipogenic differentiation of EASCs in EAT was observed between CAHD patients and non-CAHD patients according to the results of oil red O staining (data not shown). However, intervention with exosomes derived from the EAT of CAHD patients could significantly inhibit the adipogenic differentiation of EASCs (Figure 4, *P* < 0.05 vs. non-AS), suggesting that EAT-derived exosomes are a key regulatory factor in the adipogenic differentiation of EASCs.

### MiR-3064-5p Is a Key miRNA in EAT-Derived Exosomes and Targets Nnat

**Figure 5:**
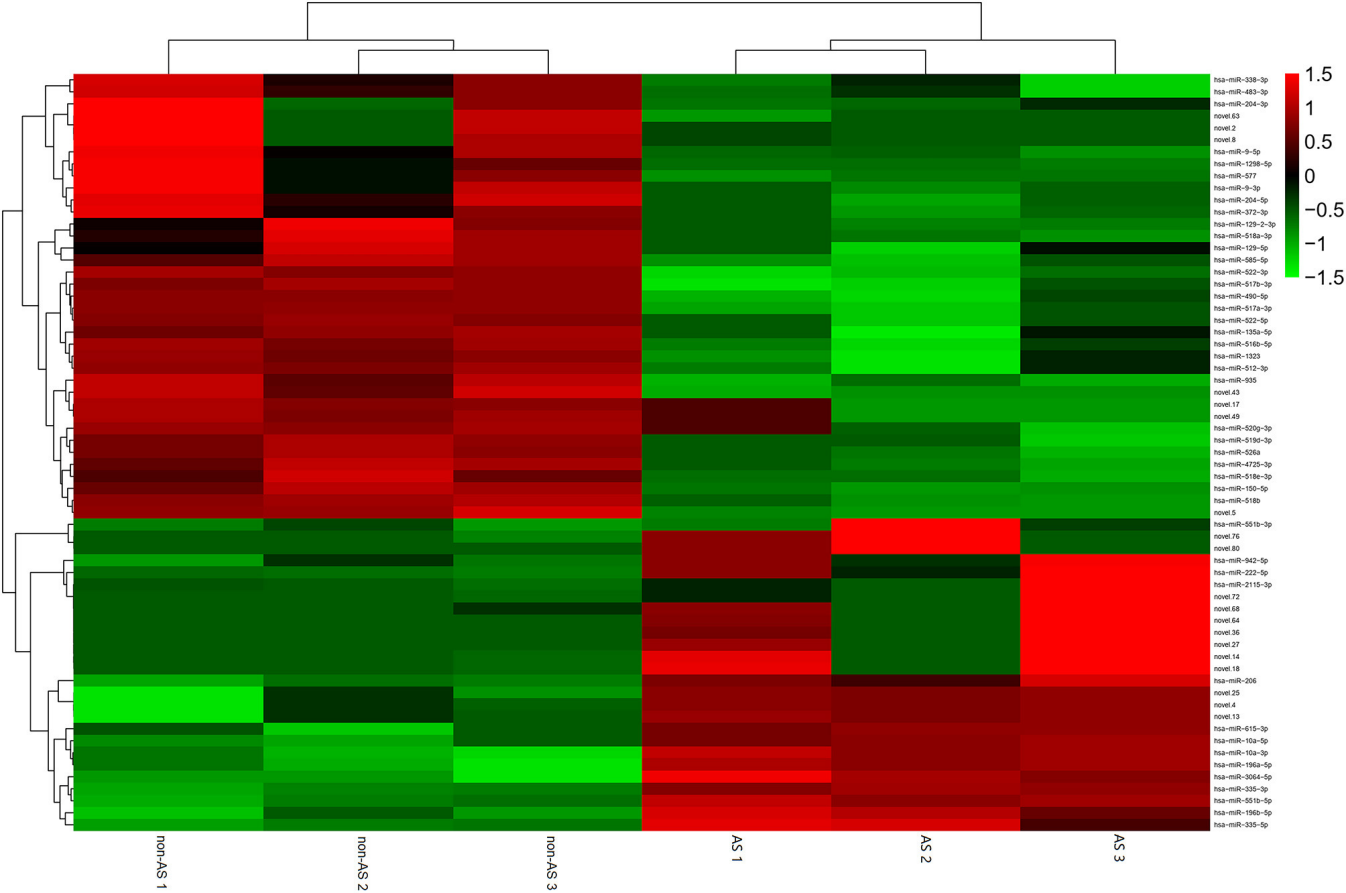
Heatmap of differential miRNAs in EAT-derived exosomes. non-AS: EAT-derived exosomes of non-CAHD patients. AS: EAT-derived exosomes of CAHD patients.

**Figure 6:**
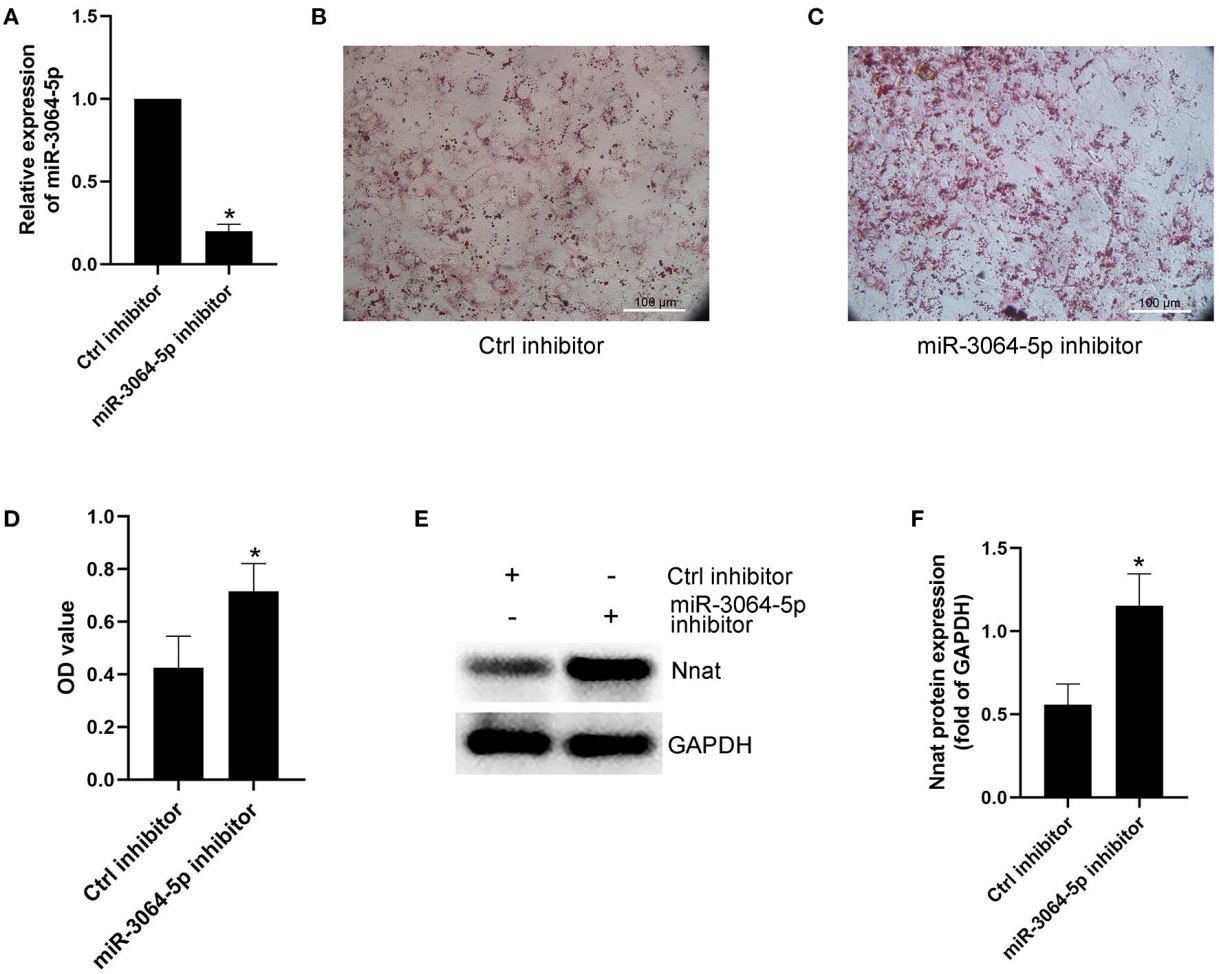
Effect of miR-3064-5p on adipogenic differentiation of EASCs. Here the EASCs were derived from the EAT of CAHD patients. **(A)** Inhibition of miR-3064-5p after adding exosome-shuttling miR-3064-5p inhibitors to the cell culture medium of EASCs. 24 h after incubation, the expression of miR-3064-5p in EASCs was detected using qPCR. **(B)** The representative image of oil red O staining in Ctrl inhibitor group. **(C)** The representative image of oil red O staining in miR-3064-5p inhibitor group. **(D)** Oil red O was extracted and absorbance was determined spectrophotometrically at 450 nm to quantify adipogenic differentiation. **(E)** The protein expression level of Nnat in groups of Ctrl inhibitor and miR-3064-5p inhibitor. **(F)** Semi quantitative analysis of western blotting in **(E)**. Data are presented as the means ± standard error of the mean for six independent experiments. ^*^P < 0.05 vs. Ctrl inhibitor.

**Figure 7:**
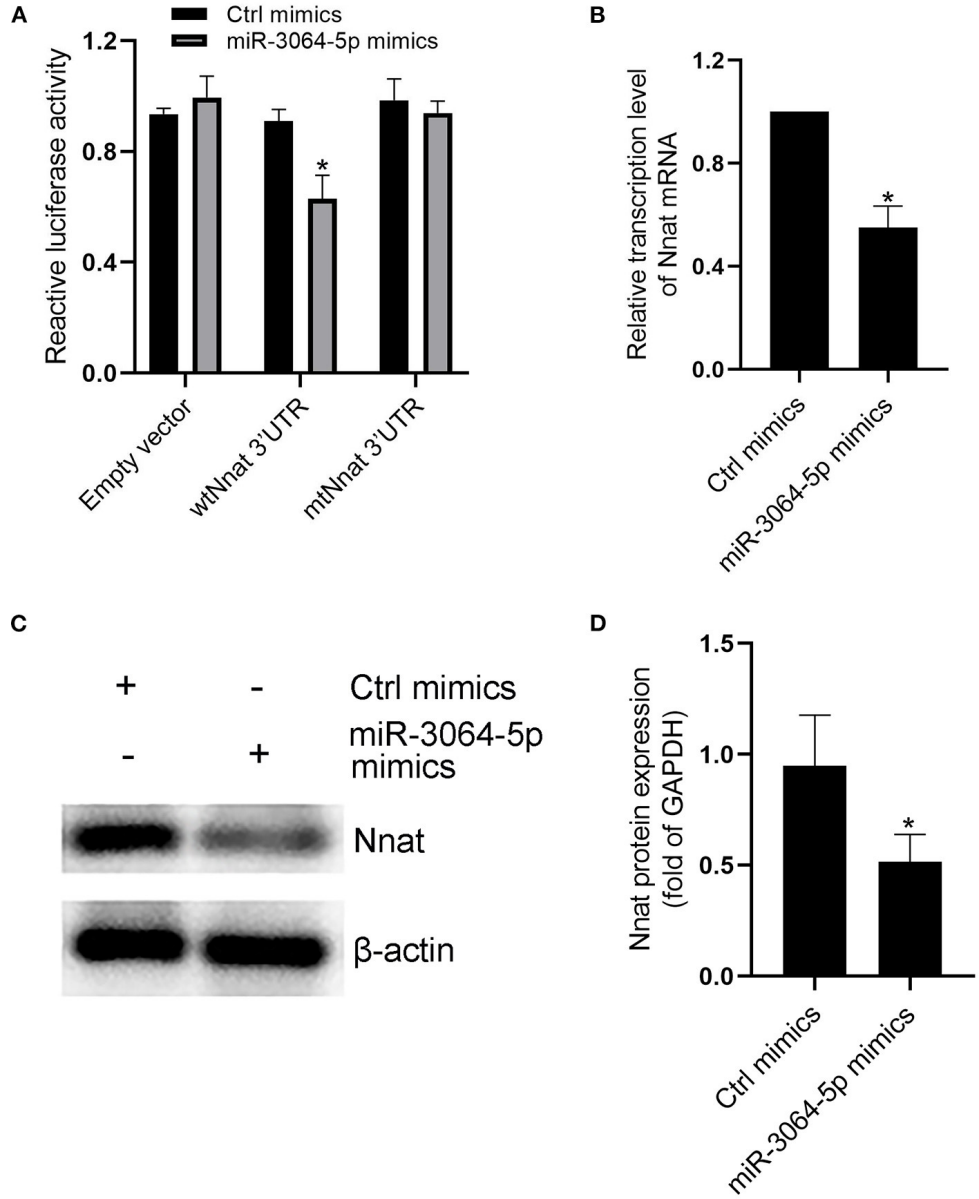
Evidences that miR-3064-5p targeting Nnat. **(A)** The reporter constructs containing the WT or Mut Nnat 3′ UTR regions were co-transfected with control mimics or miR-3064 mimics plasmid into mature adipocytes. After 24 h, firefly luciferase activity in each sample was measured and normalized to control luciferase activity. **(B)** The mRNA transcription level of Nnat after intervention of miR-3064-5p mimics. **(C)** The protein expression level of Nnat after intervention of miR-3064-5p mimics. **(D)** Semi quantitative analysis of western blotting in **(C)**. Data are presented as the means ± standard error of the mean for six independent experiments. ^*^P < 0.05 vs. Ctrl mimics.

The total RNAs of exosomes derived from EAT of CAHD patients and non-CAHD patients were extracted and subjected to miRNA sequencing. As shown in Figure 5, 63 miRNAs (fold-change ≥ 2) with significant differences in expression levels were screened. The mimics or inhibitors of these abovementioned miRNAs were synthesized and transfected into EAT-derived exosomes of CAHD patients to observe the effects of modified exosomes on the adipogenic differentiation of EASCs. The results showed that the knock-down of miR-3064-5p, which was obviously up-regulated in the exosomes derived from the EATs of CAHD patients compared with those derived from non-CAHD patients, in exosomes could significantly improve the adipogenic differentiation of EASCs (Figures 6A–D). TargetScan 7.2 was employed to determine the predicted target genes of miRNA-3064-5p, and it showed 562 target genes for miRNA-3064. Among these, we focused on Nnat, with a target score of 95. Furthermore, protein expression of Nnat was detected to be significantly up-regulated in induced EASCs after intervention with miR-3064 knock-down exosomes (Figures 6E,F). The luciferase reporter assay showed no effect on luciferase activity in cells transfected with wtNnat 3′ UTR or empty vectors (Figure 7A). In addition, compared with the cells transfected with Ctrl mimics, those transfected with miR-3064-5p mimics showed significant down-regulation in mRNA and protein expression levels of Nnat (Figures 7B–D).

### Nnat Is Silent in EASCs and Shows Low-Level Expression in EAT of CAHD Patients

**Figure 8:**
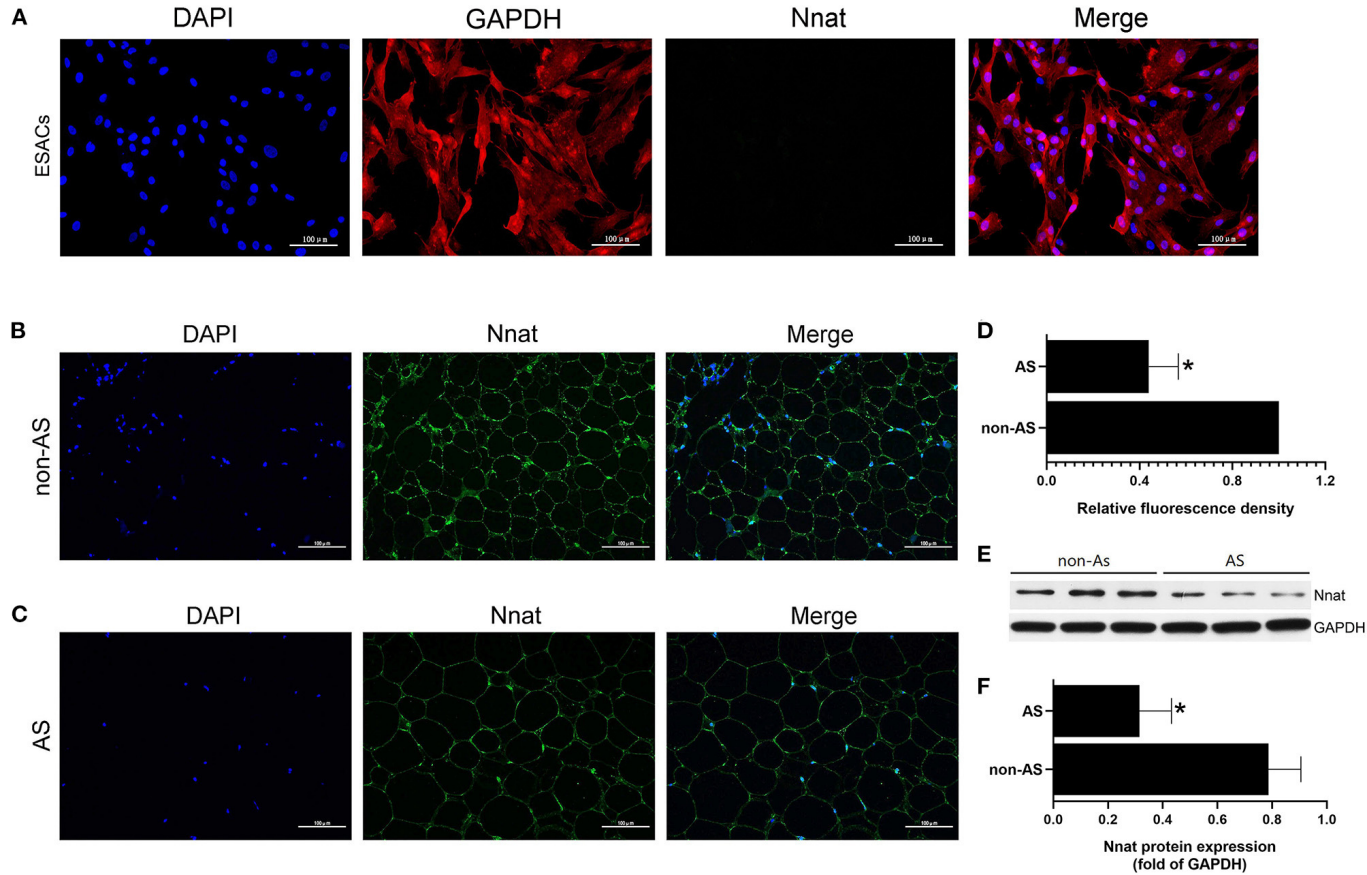
The protein expression level of Nnat in EASCs and EAT. **(A)** Immunofluorescence staining of Nnat in EASCs climbing slices. **(B)** Immunofluorescence staining of Nnat in EAT of non-CAHD patients. **(C)** Immunofluorescence staining of Nnat in EAT of CAHD patients. **(D)** Relative fluorescence density analysis of Nnat in EAT of CAHD and non-CAHD patients. **(E)** The representative western blotting bands of Nnat in EAT of CAHD and non-CAHD patients. **(F)** Semi quantitative analysis of western blotting in **(E)**. non-AS: the EAT of non-CAHD patients. AS: the EAT of CAHD patients. Data are presented as the means ± standard error of the mean for six independent experiments. ^*^P < 0.05 vs. non-AS.

The immunofluorescence double-labeling method was used to detect protein expression levels of GAPDH and Nnat in EASCs cell slides. As shown in Figure 8A, extensive expression of GAPDH was shown in EASCs, but Nnat did not show significant fluorescence, indicating silenced expression of Nnat in EASCs. Immunofluorescence staining was further performed to observe the expression level of Nnat in EAT of CAHD patients and non-CAHD patients. As shown in Figures 4B–D, the expression level of Nnat in EAT of CAHD patients was significantly down-regulated comparing with that in EAT of non-CAHD patients (*P* < 0.05). Western blotting further verified this result as shown in Figures 8E,F.

## Discussion

For a long time, adipose tissue has been considered to be an energy storage site and endocrine organ. However, in the past few decades, adipose tissue has also been found to be a rich source of mesenchymal stem cells, and is currently a research hotspot in the field of induced spontaneous regeneration and cell therapy. ASCs are easy to obtain and have a strong ability to proliferate *in vitro* and differentiate into other cell types, such as adipocytes, osteoblasts, cardiomyocytes, and hepatocytes (5, 8). Similar to the adipose tissue, abundant ASCs were also found in EAT. In this study, we found that the abundance of EASCs in EATs of CAHD patients was significantly higher than that in EATs of non-CAHD patients. We isolated human EASCs and cultured them *in vitro*, and found that there was no significant difference in the adipogenic differentiation ability of EASCs between CAHD and non-CAHD patients. Thus, we speculated that the abnormal microenvironment of EAT in CAHD patients resulting from various triggers (such as inflammation and insulin resistance) inhibited the normal differentiation of EASCs into mature adipocytes. As a result, the normal metabolism of EAT would be disturbed, leading to the dysfunction of EAT, and its autocrine and paracrine cytokines would further deteriorate the EAT microenvironment, forming a vicious circle, and aggravating the formation and progress of AS through the fat-vascular axis. However, there is a fact that CAHD patients have a thicker EAT than non-CAHD patients. One possibility is that when EASCs are induced to differentiate into adipocytes, the differentiation of EASCs into other cell types can be inhibited to a certain extent, and these cell types may be the key to CAHD. This is worth exploring in the further experiments.

Exosomes are a type of extracellular vesicle produced by cellular exocytosis, with a diameter of 30–100 nm, and are composed of lipid bilayers. These vesicles contain a wide range of degradable molecules such as proteins, lipids, and RNA, among others, which can be directly be endocytosed by target cells affecting their biological behavior (9). MicroRNA (miRNA) is the most abundant component among the contents of exosomes. MiRNA is a type of short non-coding RNA, which regulates the transcription and synthesis of proteins by interfering with mRNA transcription and translation. In adipocytes, miRNA can act on multiple targets, affecting adipocyte differentiation and metabolic homeostasis (10, 11). In EAT, miRNA may be involved in the regulation of inflammatory responses and can affect the occurrence and development of coronary artery disease (12). In this study, we cultured EAT blocks *in vitro* and isolated EAT-derived exosomes from the culture medium. We found that the adipogenic differentiation ability of EASCs after intervention with EAT-derived exosomes obtained from CAHD patients was significantly attenuated compared with that of EASCs after intervention EAT-derived exosomes from non-CAHD patients. Thus, we hypothesized that EAT-derived exosomes were involved in the regulation of adipogenic differentiation of EASCs. When a pathological microenvironment is formed in EAT, the composition and quantity of miRNAs carried by the exosomes produced by EAT changes accordingly. After being endocytosed by EASCs, the miRNAs in exosomes interfere with the expression levels of key proteins, which ultimately leads to abnormal adipogenic differentiation of EASCs.

To further explore which miRNAs play a major regulatory role, miRNA sequencing was performed to analyze the differential expression of miRNAs in EAT-derived exosomes. At present, there are relatively few research reports on miR-3064-5p, and the studies mainly focus on tumor-related fields. In our study, we noticed that miR-3064-5p was significantly up-regulated in EAT-derived exosomes from CAHD patients, and its inhibitor could obviously improve the inhibitory effect of CAHD-derived exosomes on the adipogenic differentiation of EASCs (13, 14). Further analysis showed that Nnat is the target gene of miR-3064-5p in EASCs. Exosomes modified with the miR-3064-5p inhibitor showed significant up-regulation in the expression of Nnat protein in induced EASCs. The abovementioned preliminary experimental results suggest that miR-3064-5p in EAT-derived exosomes participates in the regulation of adipogenic differentiation of EASCs by targeting Nnat.

Nnat is a gene related to neurodevelopment, and is involved in pathophysiological processes such as neurodevelopment and metabolism (15). Nnat mainly expresses in adult cerebral cortex, endocrine tissue, placenta, and adipose tissue, and its abnormal expression is associated with diabetes, obesity, and Lafora disease, which may be caused by an Nnat-mediated abnormality in Ca signaling abnormality, inflammation response, glucose exchange, or Nnat misfolding (16, 17). Yang et al. previously reported that the knockdown of Nnat expression reversed the effects of adiponectin on promoting the differentiation of 3T3-L1 cells into mature adipocytes, and inhibiting the release of inflammatory factors and oxidative stress through NF-κB signaling pathway (7). Inflammation and oxidative stress affect adipose metabolism and participate in the incidence and development of various diseases, such as AS, obesity, hypertension, and diabetes (18–21). Analysis of clinical EAT samples in this study showed that the expression of Nnat protein in EAT of CAHD patients was significantly down-regulated compared that in EAT of non-CAHD patients. Interestingly, in isolated EASCs, Nnat protein was almost not expressed and was in a silent state. We speculated that the silent state of Nnat may be the key for EASCs to maintain the characteristics of stem cells without adipogenic differentiation. Nnat activates when EASCs receive an adipogenic signal in the microenvironment, thus promoting the adipogenic differentiation of EASCs. However, the exosomes produced in the metabolically disordered EAT show miR-3064-5p over-expression, which can inhibit the protein transcriptional expression of Nnat after acting on EASCs, in turn resulting in the inability of EASCs to normally differentiate into mature adipocytes even after receiving adipogenic differentiation signals. Nevertheless, this is only a reasonable speculation, and further experimental research is still needed to verify this.

## Conclusion

In conclusion, this study found that CAHD patients contained higher levels of EASCs in EAT, and no difference in the adipogenic differentiation ability of EASCs *in vitro* was reported regardless of whether the EASCs were CAHD or non-CAHD derived. This suggested that the microenvironment of EAT was affecting the normal adipogenic differentiation of EASCs. We isolated exosomes from EAT, and confirmed that EAT-derived exosomes from CAHD patients inhibited the adipogenic differentiation of EASCs. We further reported that miR-3064-5p may be the key miRNA for EAT-derived exosomes to regulate the adipogenic differentiation of EASCs, which may play a regulatory role by targeting Nnat. In contrast, Nnat was shown to have a low expression in EAT of CAHD patients and was not expressed in EASCs, suggesting that Nnat is a key regulatory protein for the adipogenic differentiation of EASCs. However, the exact mechanism of Nnat in the regulation of adipogenesis of EASCs still needs to be further explored. In addition, future studies should also focus on the cell types derived from exosomes showing a high expression of miR-3064-5p to have a deeper understanding of the influence of the EAT microenvironment on the occurrence and development of CAHD.

## Data Availability Statement

The raw data supporting the conclusions of this article will be made available by the authors, without undue reservation.

## Ethics Statement

The studies involving human participants were reviewed and approved by Central People's Hospital of Zhanjiang Ethics Committee. The patients/participants provided their written informed consent to participate in this study.

## Author Contributions

WY and HT designed the study, interpreted data, and wrote the manuscript. WY, HT, KT, and HH performed laboratory measurements and analyzed data. KT and HH interpreted data and critically revised the manuscript. SO and JW collected clinical samples and assisted in the experiments. All authors contributed to the article and approved the submitted version.

## Conflict of Interest

The authors declare that the research was conducted in the absence of any commercial or financial relationships that could be construed as a potential conflict of interest.

## Publisher's Note

All claims expressed in this article are solely those of the authors and do not necessarily represent those of their affiliated organizations, or those of the publisher, the editors and the reviewers. Any product that may be evaluated in this article, or claim that may be made by its manufacturer, is not guaranteed or endorsed by the publisher.

## References

[1] Ross R. Atherosclerosis-An inflammatory disease. N Engl J Med. (1999) 340:115–26. 10.1056/NEJM199901143400207 9887164

[2] Wu Y Zhang A Hamilton DJ Deng T. Epicardial fat in the maintenance of cardiovascular health. Methodist Debakey Cardiovasc J. (2017) 13:20–4. 10.14797/mdcj-13-1-20 28413578 PMC5385790

[3] Furuhashi M Fuseya T Murata M Hoshina K Ishimura S Mita T. Local production of fatty acid-binding protein 4 in epicardial/perivascular fat and macrophages is linked to coronary atherosclerosis. Arterioscler Thromb Vasc Biol. (2016) 36:825–34. 10.1161/ATVBAHA.116.307225 27013610

[4] Mancio J Azevedo D Saraiva F Azevedo AI Pires-Morais G Leite-Moreira A. Epicardial adipose tissue volume assessed by computed tomography and coronary artery disease: a systematic review and meta-analysis. Eur Heart J Cardiovasc Imaging. (2018) 19:3267. 10.1093/ehjci/jex314 29236951

[5] Ansaldo AM Montecucco F Sahebkar A Dallegri F Carbone F. Epicardial adipose tissue and cardiovascular diseases. Int J Cardiol. (2019) 278:254–60. 10.1016/j.ijcard.2018.09.089 30297191

[6] Wystrychowski W Patlolla B Zhuge Y Neofytou E Robbins RC Beygui RE. Multipotency and cardiomyogenic potential of human adipose-derived stem cells from epicardium, pericardium, and omentum. Stem Cell Res Ther. (2016) 7:84. 10.1186/s13287-016-0343-y 27296220 PMC4907285

[7] Yang W Yuan W Peng X Wang M Xiao J Wu C. PPAR γ/Nnat/NF-κB axis involved in promoting effects of adiponectin on preadipocyte differentiation. Mediators Inflamm. (2019) 2019:5618023. 10.1155/2019/5618023 31871428 PMC6906841

[8] Matsuda M Shimomura I Sata M Arita Y Nishida M Maeda N. Role of adiponectin in preventing vascular stenosis THE MISSING LINK OF ADIPO-VASCULAR AXIS. J Biol Chem. (2003) 67:37487–91. 10.1074/jbc.M206083200 12138120

[9] Zhang Y Bi J Huang J Tang Y Du S Li P. Exosome: a review of its classification, isolation techniques, storage, diagnostic and targeted therapy applications. Int J Nanomedicine. (2020) 15:6917–34. 10.2147/IJN.S264498 33061359 PMC7519827

[10] Lorente-Cebrián S González-Muniesa P Milagro FI Martínez JA. MicroRNAs and other non-coding RNAs in adipose tissue and obesity: emerging roles as biomarkers and therapeutic targets. Clin Sci (Lond). (2019) 133:23–40. 10.1042/CS20180890 30606812

[11] Arias N Aguirre L Fernández-Quintela A González M Lasa A Miranda J. MicroRNAs involved in the browning process of adipocytes. J Physiol Biochem. (2016) 72:509–21. 10.1007/s13105-015-0459-z 26695012

[12] Huang W Wu X Xue Y Zhou Y Xiang H Yang W. MicroRNA-3614 regulates inflammatory response via targeting TRAF6-mediated MAPKs and NF-κB signaling in the epicardial adipose tissue with coronary artery disease - ScienceDirect. Int J Cardiol. (2020) 324:152–64. 10.1016/j.ijcard.2020.09.045 32950591

[13] Zhang P Ha M Li L Huang X Liu C. MicroRNA-3064-5p sponged by MALAT1 suppresses angiogenesis in human hepatocellular carcinoma by targeting the FOXA1/CD24/Src pathway. FASEB J. (2020) 34:66–81. 10.1096/fj.201901834R 31914639

[14] Wang S Ping M Song B Guo Y Li Y Jia J. Exosomal CircPRRX1 enhances doxorubicin resistance in gastric cancer by regulating MiR-3064-5p/PTPN14 signaling. Yonsei Med J. (2020) 61:750–61. 10.3349/ymj.2020.61.9.750 32882759 PMC7471080

[15] Pitale PM Howse W Gorbatyuk M. Neuronatin protein in health and disease. J Cell Physiol. (2016) 232:477–81. 10.1002/jcp.25498 27442611

[16] Joseph RM. Neuronatin gene: imprinted and misfolded: studies in Lafora disease, diabetes and cancer may implicate NNAT-aggregates as a common downstream participant in neuronal loss. Genomics. (2014) 103:183–8. 10.1016/j.ygeno.2013.12.001 24345642

[17] Ka HI Han S Jeong AL Lee S Yong HJ Boldbaatar A. Neuronatin is associated with anti-inflammatory role in the white adipose tissue. J Microbiol Biotechnol. (2017) 27:1180–8. 10.4014/jmb.1702.02049 28335587

[18] Victorio JA Davel AP. Perivascular adipose tissue oxidative stress on the pathophysiology of cardiometabolic diseases. Curr Hypertens Rev. (2019) 16:192–200. 10.2174/1573402115666190410153634 30968777

[19] Lefranc C Friederich-Persson M Braud L Palacios-Ramirez R Karlsson S Boujardine N. MR (Mineralocorticoid Receptor) induces adipose tissue senescence and mitochondrial dysfunction leading to vascular dysfunction in obesity. Hypertension. (2019) 73:458–68. 10.1161/HYPERTENSIONAHA.118.11873 30624990

[20] Kong L Zhou Y Chen D Ruan C Gao P. Decrease of perivascular adipose tissue browning is associated with vascular dysfunction in spontaneous hypertensive rats during aging. Front Physiol. (2018) 9:400. 10.3389/fphys.2018.00400 29720945 PMC5915562

[21] Nacci C Leo V Benedictis LD Potenza MA Sgarra L Salvia MAD. Infliximab therapy restores adiponectin expression in perivascular adipose tissue and improves endothelial nitric oxide-mediated vasodilation in mice with type 1 diabetes. Vascul Pharmacol. (2016) 87:83–91. 10.1016/j.vph.2016.08.007 27565410

