# Supplementary figures and images for: MiR-3064 in Epicardial Adipose-Derived Exosomes Targets Neuronatin to Regulate Adipogenic Differentiation of Epicardial Adipose Stem Cells
Source: Front Cardiovasc Med. 2021 Aug 18;8:709079. doi: 10.3389/fcvm.2021.709079 (PMC8416507; doi:10.3389/fcvm.2021.709079)

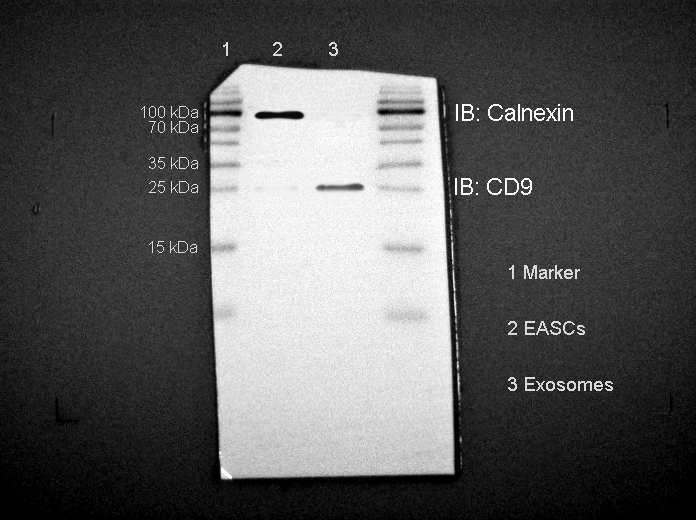

Supplement: Supplementary file 1 [file Image_1.TIF]

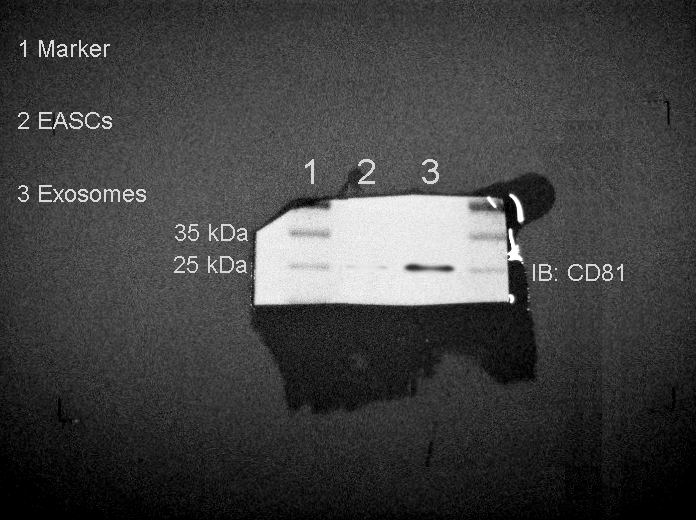

Supplement: Supplementary file 2 [file Image_2.PNG]

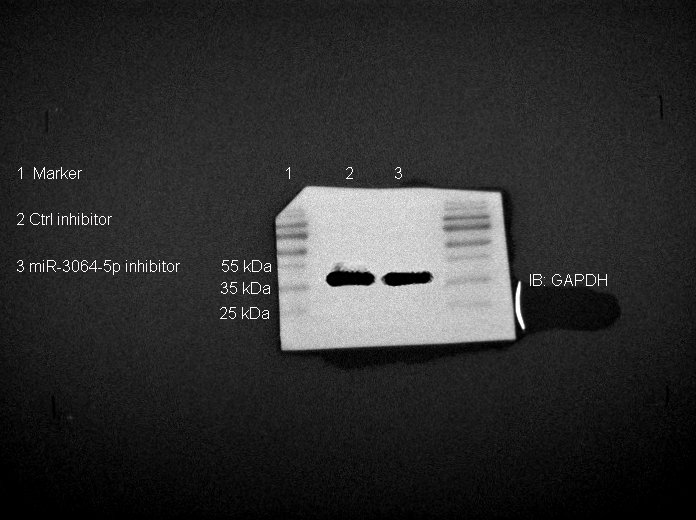

Supplement: Supplementary file 3 [file Image_3.TIF]

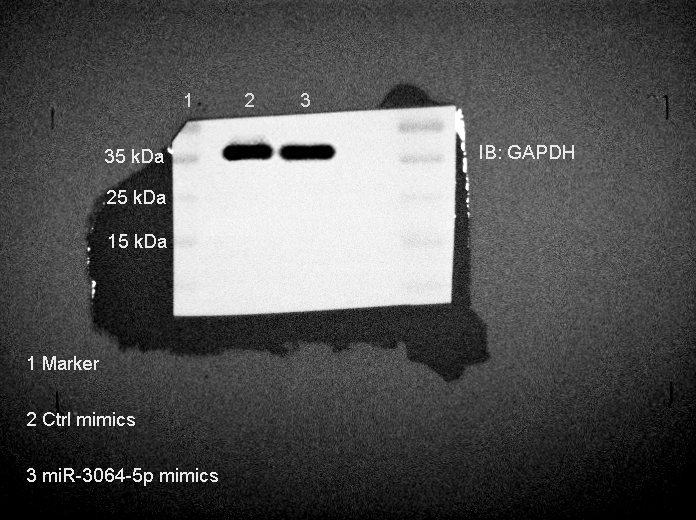

Supplement: Supplementary file 4 [file Image_4.TIF]

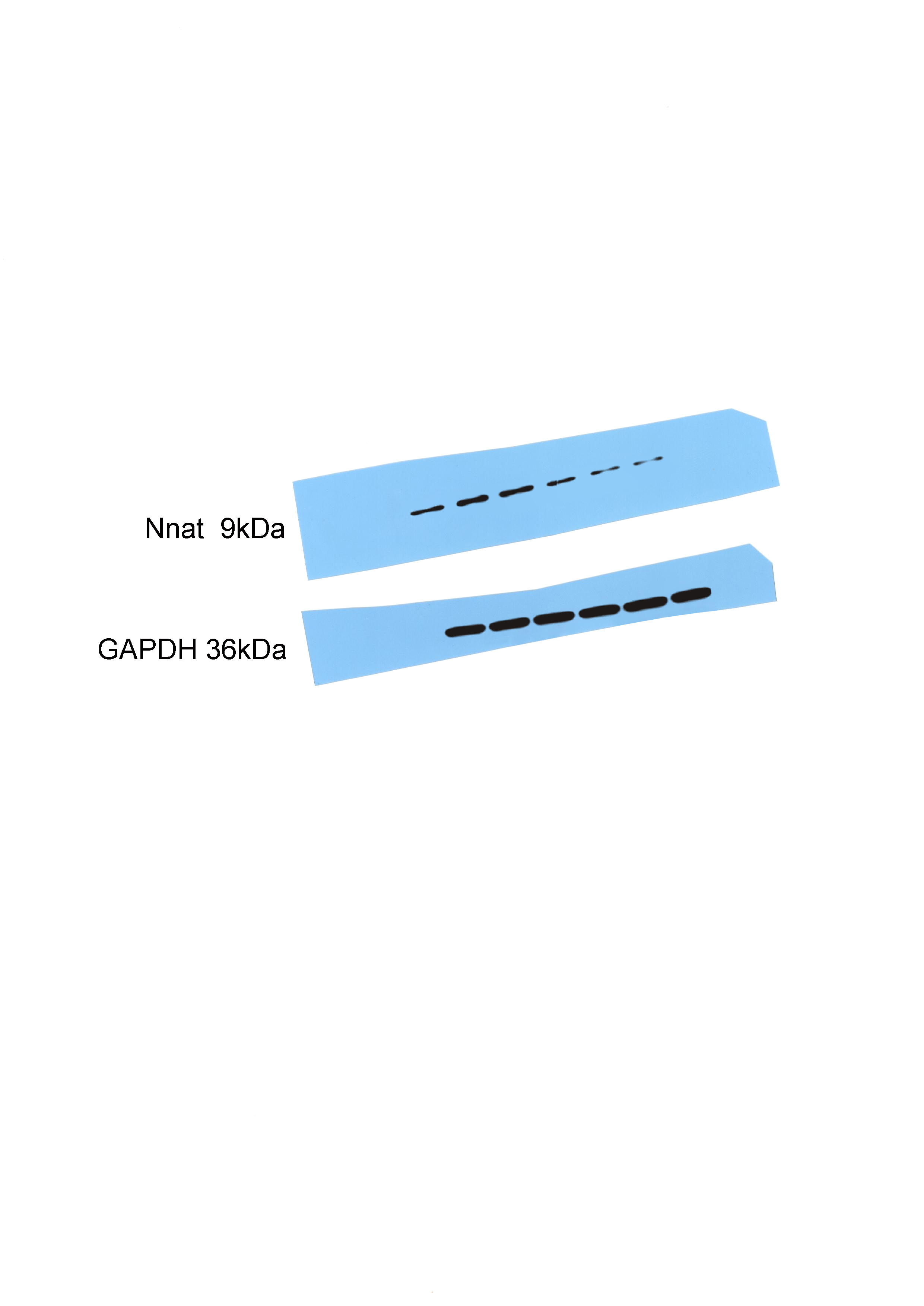

Supplement: Supplementary file 5 [file Image_5.TIF]

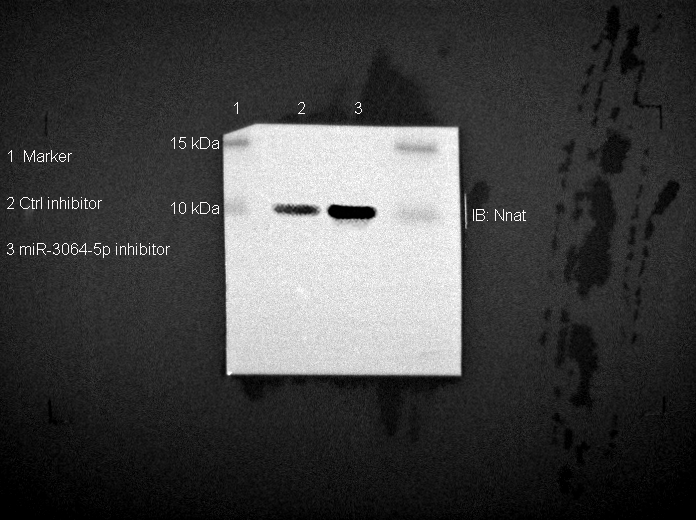

Supplement: Supplementary file 6 [file Image_6.TIF]

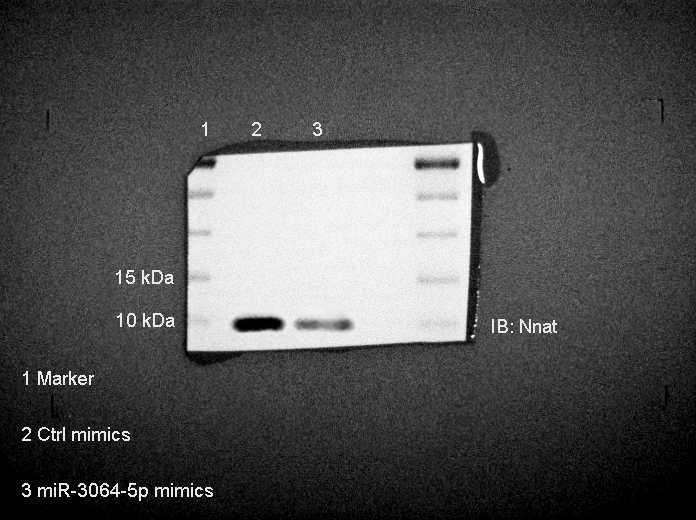

Supplement: Supplementary file 7 [file Image_7.TIF]
